# Successful disease-specific induced pluripotent stem cell generation from patients with kidney transplantation

**DOI:** 10.1186/scrt89

**Published:** 2011-12-06

**Authors:** Tayaramma Thatava, Adam S Armstrong, Josep Genebriera De Lamo, Ramakrishna Edukulla, Yulia Krotova Khan, Toshie Sakuma, Seiga Ohmine, Jamie L Sundsbak, Peter C Harris, Yogish C Kudva, Yasuhiro Ikeda

**Affiliations:** 1 Department of Molecular Medicine, Mayo Clinic, Rochester, MN55905, USA; 2 Human Cell Therapy Laboratory, Mayo Clinic, Rochester, MN 55905, USA; 3 Dermatology, Mayo Clinic, Rochester, MN 55905, USA; 4 Nephrology and Hypertension Research, Mayo Clinic, Rochester, MN 55905, USA; 5 Division of Endocrinology, Mayo Clinic, Rochester, MN 55905, USA

## Abstract

**Introduction:**

End-stage renal disease (ESRD) is a major public health problem. Although kidney transplantation is a viable therapeutic option, this therapy is associated with significant limitations, including a shortage of donor organs. Induced pluripotent stem (iPS) cell technology, which allows derivation of patient-specific pluripotent stem cells, could provide a possible alternative modality for kidney replacement therapy for patients with ESRD.

**Methods:**

The feasibility of iPS cell generation from patients with a history of ESRD was investigated using lentiviral vectors expressing pluripotency-associated factors.

**Results:**

In the present article we report, for the first time, generation of iPS cells from kidney transplant recipients with a history of autosomal-dominant polycystic kidney disease (ADPKD), systemic lupus erythematosus, or Wilms tumor and ESRD. Lentiviral transduction of OCT4, SOX2, KLF4 and c-MYC, under feeder-free conditions, resulted in reprogramming of skin-derived keratinocytes. Keratinocyte-derived iPS cells exhibited properties of human embryonic stem cells, including morphology, growth properties, expression of pluripotency genes and surface markers, spontaneous differentiation and teratoma formation. All iPS cell clones from the ADPKD patient retained the conserved W3842X mutation in exon 41 of the *PKD1 *gene.

**Conclusions:**

Our results demonstrate successful iPS cell generation from patients with a history of ESRD, *PKD1 *gene mutation, or chronic immunosuppression. iPS cells from autosomal kidney diseases, such as ADPKD, would provide unique opportunities to study patient-specific disease pathogenesis *in vitro*.

## Introduction

The prevalence of chronic kidney disease and end-stage renal disease (ESRD) is increasing worldwide [1]. Simultaneously, the total Medicare cost of ESRD has risen from $12.2 billion in 2000 to $39.5 billion in 2010 [1]. ESRD is incurable, requiring hemodialysis or preferably renal transplantation. These therapies are associated with considerable limitations, however, including the shortage of available donor organs and a lifelong immunosuppressive regimen [2]. Moreover, despite significant improvement in 1-year kidney allograft survival, the rate of chronic graft loss after the first year remains substantial [3].

The most common causes of ESRD in the United States are diabetes and hypertension [4], while the incidence of nondiabetic ESRD, such as glomerular diseases and cystic diseases, are increasing. Autosomal-dominant polycystic kidney disease (ADPKD) is the most common life-threatening, lethal genetic disease, affecting approximately 7 million people worldwide. Mutations in the *PKD1 *and *PKD2 *genes are responsible for ADPKD in 85% and 15% of patients, respectively. ADPKD is the leading hereditary cause of ESRD, accounting for approximately 4% of ESRD [5]. Wilms tumor (WT) or nephroblastoma is a rare kidney cancer, and is responsible for 95% of all kidney tumors in children [6]. Although the risk of ESRD is low for the majority of WT patients, ESRD can occur from chemotherapy-induced nephrotoxicity or radiation-induced obstructive uropathy. Those with WT-aniridia syndrome or associated genitourinary anomalies (hypospadias or cryptorchism) are at higher risk of ESRD, and therefore require indefinite screening for renal function [7]. Systemic lupus erythematosus (SLE) is an autoimmune disease with inflammation-mediated multiorgan damage. Kidney is one of the main target organs in SLE, and lupus nephritis is a major cause of morbidity and mortality. Approximately 10% of patients with SLE develop ESRD [8]. In SLE patients with kidney transplant, recurrence of the disease in the graft is frequently observed [9].

Stem cell-based regenerative medicine approaches hold great promise to treat patients with degenerative diseases. Successful replacement, or augmentation, of the function of damaged renal cells by stem cells would provide a novel cell-based therapy for renal diseases. Although adult stem cells, such as bone marrow stem cells, can differentiate into renal resident cells and participate in kidney regeneration [10], their engraftment into injured tubules and development into functional renal tissues are not sufficient to repair acute renal injury [11,12]. Accordingly, pluripotent stem cell-based kidney tissue engineering has attracted considerable attention. Although embryonic stem (ES) cells have provided a unique platform for pluripotent stem cell-based regenerative medicine applications, their widespread use in the clinic is restricted by ethical issues and allogenic mismatch.

Generation of induced pluripotent stem (iPS) cells from adult somatic cells from mouse fibroblasts was first reported through retroviral transduction of *Oct4*, *Sox2*, *Klf4 *and c-*Myc *genes [13]. Subsequently, human iPS cells were generated from human fibroblasts through introduction of a set of stemness factors: OCT4, SOX2, KLF4 and c-MYC [14,15], or OCT4, SOX2, NANOG and LIN28 [16]. Since iPS cells resemble ES cells in their ability to generate cells of three germ layers, patient-specific iPS cells offer potential solutions for the ethical and histo-incompatibility issues of ES cells. Moreover, disease-specific iPS cell derivation and differentiation into relevant tissues/organs could provide unique opportunities to model disease progression *in vitro*, to screen patient-specific drugs and, ultimately, to enable immunosuppression-free cell replacement therapy. Although autologous iPS cells have been generated from patients with various diseases/disorders [17-23], the feasibility and reproducibility of iPS cell derivation from kidney diseases, such as ADPKD, has remained elusive.

In the present study, we recruited patients who had a clinical history of ESRD due to ADPKD, SLE or WT/obstructive uropathy, kidney transplant and maintenance on an immunosuppressive regimen, and examined the feasibility of iPS cell derivation. We were able to reprogram skin-derived keratinocytes into patient-specific iPS cells. These kidney disease-specific iPS cell clones expressed pluripotency markers and differentiated into cells of three germ layers *in vitro *and *in vivo*, verifying their pluripotency. Our results therefore demonstrated successful iPS cell generation from patients with a history of ESRD, *PKD1 *gene mutation or chronic immunosuppression. iPS cells from autosomal kidney diseases, such as ADPKD, would enable studies of patient-specific renal disease pathogenesis at a cellular level, while autologous iPS cells could be used for novel kidney replacement therapy.

## Materials and methods

All studies were approved by the Mayo Institutional Review Board and Institutional Animal Care and Use Committee. We recruited patients from the kidney transplant program. All patients provided written informed consent for this research study. Patient information is summarized in Table 1.

**Table 1:** Patient information

Patient	Immunosuppressants	Daily dose (mg)	Length of exposure (years)
WT	Mycophenolate/prednisone/tacrolimus	3.5/10/1,000	4
SLE	Mycophenolate/prednisone/tacrolimus	3/7/1,000	11
ADPKD	Mycophenolate/tacrolimus	3.5/2,000	1

ADPKD, autosomal-dominant polycystic kidney disease; SLE, system lupus erythematosus; WT, Wilms tumor.

### Derivation of patients' fibroblasts and keratinocytes under xeno-free conditions

Skin biopsy of an 8 mm punch was obtained and processed to derive dermal keratinocytes and fibroblasts; briefly, tissue was submerged in enough dispase solution (Invitrogen catalog number 17105-041; Invitrogen, Grand Island, NY, USA) for either 16 to 21 hours at 4°C or 3 to 4 hours at 37°C for separation of epidermis from dermis. Once separated, epidermis was collected into a vial containing recombinant trypsin/ethylenediamine tetraacetic acid (catalog number R-009-50; Invitrogen) for separation of fibroblasts and keratinocytes, and incubated at 37°C for 15 to 30 minutes. Defined trypsin inhibitor (catalog number R-007-100; Invitrogen) was used to neutralize the trypsin/ethylenediamine tetraacetic acid. Human keratinocytes and fibroblasts were cultured in an animal-origin-free media. EpiLife Medium (catalog number M-EPI-500-CA; Invitrogen) with Supplement S7 (catalog number S-017-5; Invitrogen) was used for human keratinocytes, while fibroblast medium (FM-acf, catalog number 2321; Sciencell Research Laboratories, Carlsbad, CA, USA) was used for fibroblasts. Dermal fibroblasts and keratinocytes were cultured on collagen-coated flasks (coating matrix, catalog number R-011-K; Invitrogen) and were plated for reprogramming or frozen using Synth-a-Freeze (catalog number R-005-50; Invitrogen).

### Generation of patient-derived iPS cells under feeder-free conditions

Dermal keratinocytes were reprogrammed to generate human iPS cell clones as described previously, with minor modifications [24,25]. Briefly, 20% confluent keratinocytes were transduced with OCT3/4-expressing, SOX2-expressing, KLF4-expressing and c-MYC-expressing lentiviral vectors each at a multiplicity of infection of 5 in EpiLife medium. After overnight viral infection, cells were fed with fresh complete EpiLife medium. Culture supernatants were replaced daily with EpiLife medium until 4 days after vector infection. At day 5, the medium was replaced with a feeder-free iPS cell medium, which contained HEScGRO (catalog number SCM020; Millipore, Billerica, MA, USA) with 25% mTeSR1 medium (catalog number 05850; Stem Cell Technologies, Vancouver, BC, Canada) and antibiotics, but no additional basic fibroblast growth factor. Cells were fed with fresh HEScGRO-based iPS cell medium every 2 days. Putative iPS cell colonies were observed within 2 weeks after viral vector transduction and propagated on matrigel-coated plates, as described previously [25]. Human keratinocytes were also infected with lentiviral vectors expressing the three factors OCT4, SOX2 and KLF4, without c-MYC, for possible c-MYC-free reprogramming.

### Immunocytochemistry and alkaline phosphatase staining

Undifferentiated patient-specific iPS cell clones were fixed for 20 minutes at room temperature in 4% paraformaldehyde in PBS and then permeabilized with Triton X-100 for 30 minutes. After 30 minutes of blocking with 5% fetal bovine serum in PBS with 0.1% Tween-20, cells were stained with primary antibodies overnight at 4°C and, after PBS wash, incubated with secondary antibodies (1 hour at room temperature at a dilution of 1:200). Nuclei were counterstained with 4,6-diamidino-2-phenylindole (1:500; Sigma, St. Louis, MO, USA). Primary and secondary antibodies used for characterization of iPS cells are listed in Supplementary Table S1 in Additional file 1. After staining, cells were analyzed using confocal laser-scanning microscopy (LSM 510 confocal scanning laser system; Zeiss, Thornwood, NY, USA). For detection of alkaline phosphatase activity, the alkaline phosphatase detection kit (Millipore, Billerica, MA, USA) was used. Briefly, human iPS cells in culture for 5 days were fixed with 4% paraformaldehyde for 2 minutes at room temperature. The cells were rinsed with TBST buffer (20 mM Tris-HCl, pH 7.4, 0.15 NaCl, 0.05% Tween-20) and stained with naphthol/fast red violet solution (mixture of fast red violet, naphthol AS-BI phosphate solution and water in a 2:1:1 ratio) for 15 minutes in the dark. Cells were rinsed with TBST and covered with PBS; alkaline phosphatase-positive cells were imaged using a light microscope.

### RNA isolation and RT-PCR analysis

### Spontaneous differentiation

iPS cell clones were dissociated using collagenase type IV (catalog number 07909; Stem Cell Technologies) and then suspended cell clumps were cultured in basal HEScGRO medium (SCM 021) in low-adhesion plates. Embryoid bodies were formed in suspension, initially cultured for approximately 7 to 14 days, and then transferred to knockout DMEM with 20% fetal bovine serum, differentiated as a monolayer for another 10 to 14 days. For evaluation of the presence of cells of three germ layers, differentiated cells were fixed and stained with the antibodies for immunofluorescence analysis. Primary antibodies and secondary antibodies are listed in Supplementary Table S1 in Additional file 1.

### Teratoma formation and analysis

Undifferentiated WT, SLE and ADPKD iPS cell clones were manually detached by collagenase type IV treatment and injected subcutaneously into each flank of 8-week-old to 10-week-old SCID-Beige mice. Tumors were observed 4 to 6 weeks after injection, and collected 3 to 5 months after injection. Tumor samples were processed by 10 μm cryosectioning, and stained H & E tissue sections were analyzed.

### DNA isolation and sequencing

Total DNA was isolated from ADPKD-iPS cells using PureLink™ Genomic DNA kits (catalog number 25-1012; Invitrogen). The ADPKD patient was previously mutation characterized (family 300004) [26]. To characterize the W3842X mutation in the iPS cells, exon 41 was amplified with flanking primers and conventionally sequenced as previously described [26].

## Results

### Isolation and culture of patient-specific fibroblasts, keratinocytes and generation of iPS cells

The patients with a history of renal diseases including ADPKD, WT and SLE were from different age groups, and were on various doses of immunosuppressive drugs (mycophenolate, prednisone and tacrolimus) (Table 1). Skin biopsies obtained from patients were processed under animal-component-free conditions and keratinocytes and fibroblasts were cultured in serum-free medium. Undifferentiated keratinocytes could be expanded over 10 passages, whereas fibroblasts stopped propagation after a few passages, most probably due to cellular senescence. We therefore used keratinocytes for reprogramming. Keratinocytes were transduced with lentiviral vectors expressing four pluripotency factors OCT4, SOX2, KLF4 and c-MYC each at a multiplicity of infection of 5. Approximately five to 20 iPS cell-like colonies formed in 10^5 ^infected cells. No iPS cell-like colonies were formed in cells transduced with three factors without c-MYC. iPS cell-like colonies were handpicked, based on morphological criteria such as compact colonies, high nucleus-to-cytoplasm ratios and prominent nucleoli, and were expanded on Matrigel-coated plates under feeder-free conditions. We identified patient-specific iPS cells derived from the WT patient as WT-iPS cells, those derived from the SLE patient as SLE-iPS cells and those derived from the ADPKD patient as ADPKD-iPS cells.

### Expression of pluripotency markers in patient-specific iPS cell clones

**Figure 1:**
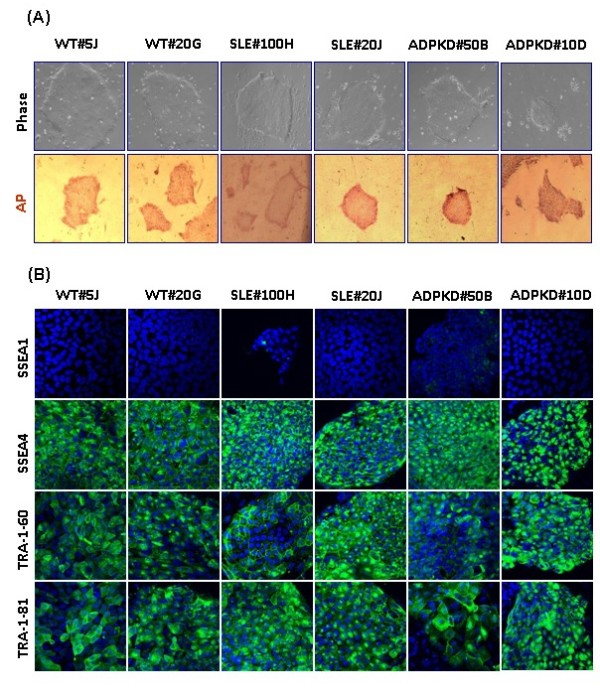
**Characterization of human induced pluripotent stem cells generated from patient-specific keratinocytes**. Patient-specific induced pluripotent stem (iPS) cell clones were generated from keratinocytes of Wilms tumor (WT), systemic lupus erythematosus (SLE) and autosomal-dominant polycystic kidney disease (ADPKD) patients by ectopic expression of lentiviral vectors expressing *OCT3/4*, *SOX2*, *KLF4 *and *c-MYC*. iPS cell clones from keratinocytes of the WT patient were termed WT-iPS, similarly for SLE-iPS and ADPKD-iPS. Two clones each were analyzed for human embryonic stem (ES) characteristics (WT#5J, WT#20G, SLE#100H, SLE#20J, ADPKD#50B and ADPKD#10D). WT-iPS, SLE-iPS and ADPKD-iPS clones were generated and maintained under feeder-free conditions, exhibiting morphology similar to human ES cells. **(A) **All of the iPS cell clones expressed alkaline phosphatase (AP). **(B) **WT-iPS, SLE-iPS and ADPKD iPS clones expressed cell surface markers SSEA4, TRA-1-60 and TRA-1-81, whereas no notable staining was observed for SSEA1. The nucleus was revealed by counterstaining with 4,6-diamidino-2-phenylindole. Phase-contrast images and AP images are 10× magnification. Immunofluorescence images were obtained at 40× magnification.

**Figure 2:**
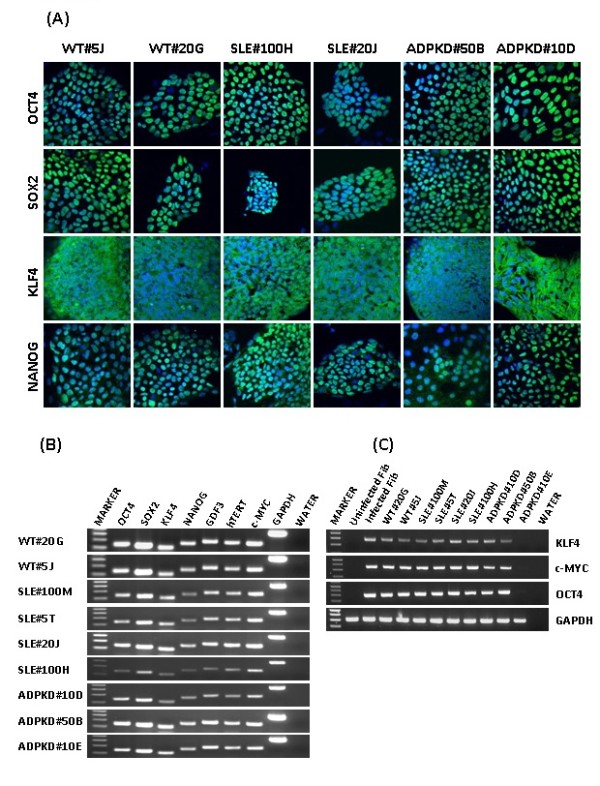
**Patient-specific induced pluripotent stem cell clones were analyzed for pluripotency-associated gene expression**. **(A) **Wilms tumor (WT)-induced pluripotent stem (iPS) cell, systemic lupus erythematosus (SLE)-iPS cell and autosomal-dominant polycystic kidney disease (ADPKD)-iPS cell clone expression of stemness-associated genes was analyzed by immunofluorescence and RT-PCR analysis. WT-iPS, SLE-iPS and ADPKD-iPS clones expressed high levels of OCT4, SOX2, KLF4 and NANOG. Immunofluorescence images were obtained at 40× magnification. **(B) **Total cellular RNA from WT-iPS, SLE-iPS and ADPKD-iPS clones was isolated and analyzed for endogenous pluripotency genes by RT-PCR. WT-iPS, SLE-iPS and ADPKD-iPS clones expressed gene transcripts of OCT4, SOX2, KLF4, NANOG, GDF3, hTERT and c-MYC. **(C) **For detection of silencing of exogenous pluripotency genes, BJ fibroblasts were infected with lentiviral vectors expressing OCT4, SOX2, KLF4 and c-MYC. Three days after viral vector transduction, RNA was isolated from infected fibroblasts, control uninfected fibroblasts, and WT-iPS, SLE-iPS and ADPKD-iPS clones. RT-PCR was performed for transgenes KLF4, c-MYC and OCT4. GAPDH gene transcript was amplified as an internal RNA control. No template (water) samples were included as controls.

Putative patient-specific iPS cell clones cultured under feeder-free and serum-free conditions exhibited morphology similar to that of human ES cells, characterized by large nuclei and scant cytoplasm, with distinct borders (Figure 1A). All of the patient-specific iPS cell clones were positive for alkaline phosphatase (Figure 1A). WT-iPS, SLE-iPS and ADPKD-iPS cell clones were verified for the presence of cell surface markers SSEA-4, TRA-1-60 and TRA-1-81 (Figure 1B). Similar to human ES cells, these clones were negative for SSEA-1 expression. WT-iPS, SLE-iPS and ADPKD-iPS cell clones also expressed pluripotency genes OCT4, SOX2, KLF4 and NANOG by immunofluorescence (Figure 2A). In contrast, isotype control staining showed no notable signals (Supplementary Figure S1 in Additional file 2). RT-PCR analyses of total cellular RNA further confirmed expression of pluripotency-associated genes, including OCT3/4, SOX2, GDF3, telomerase (TERT), KLF4, c-MYC and NANOG in the WT-iPS, SLE-iPS and ADPKD-iPS cell clones (Figure 2B). We then assessed whether expression of the vector transgenes was silenced in derived iPS cell clones. RT-PCR analysis was performed to detect OCT4-transgene-specific, KLF4-transgene-specific and c-MYC-transgene-specific transcripts. The presence of exogenous pluripotency genes was evident in most of the clones. One of the ADPKD-iPS cell clones exhibited silencing of exogenous genes (Figure 2C). Morphology and expression of pluripotent genes indicated the establishment of patient-specific iPS cell clones from keratinocytes.

### Pluripotency validated through spontaneous differentiation and teratoma formation

**Figure 3:**
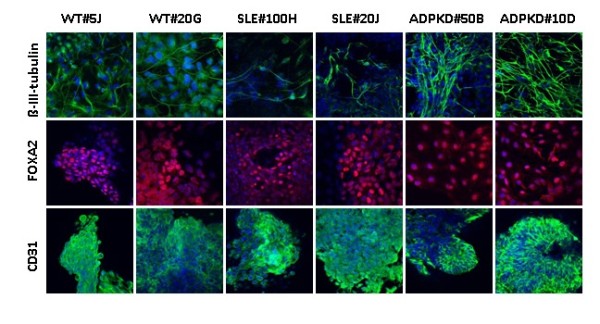
**Induced pluripotent stem cell clone spontaneous differentiation into cells of embryonic germ layers**. Wilms tumor (WT)-induced pluripotent stem (iPS) cell, systemic lupus erythematosus (SLE)-iPS cell and autosomal-dominant polycystic kidney disease (ADPKD)-iPS cell clones were allowed to spontaneously differentiate as embryoid bodies in suspension, followed by adherent culture for 10 to 14 days. Differentiated cells were immunostained for markers of ectoderm, endoderm and mesoderm lineages β-III-tubulin (green), FOXA2 (red) and CD31 (PECAM-1) (green), respectively. Nuclei were stained with 4,6-diamidino-2-phenylindole. All images were obtained at 40× magnification.

**Figure 4:**
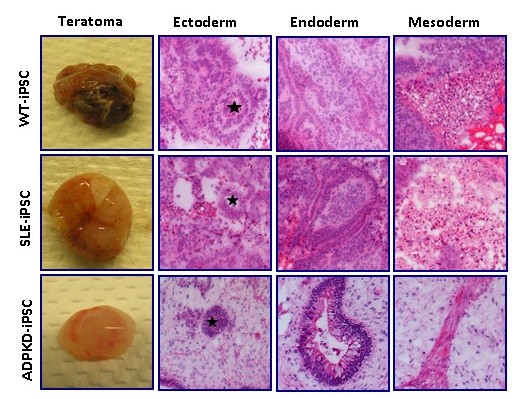
**Wilms tumor and systemic lupus erythematosus induced pluripotent stem cells form teratoma in immunodeficient mice**. Wilms tumor (WT)-induced pluripotent stem (iPS) cell, systemic lupus erythematosus (SLE)-iPS cell and autosomal-dominant polycystic kidney disease (ADPKD)-iPS cell clones were injected subcutaneously into SCID-Beige mice. Tumors were detected from the site of injection and harvested after 3 to 5 months, examined for the presence of cells of three embryonic germ layers. H & E-stained teratoma showed multiple differentiated tissues including cells of ectoderm, neuronal rosette-like structures (indicated *), endoderm (gut tube-like structures, intestinal epithelial cells) and mesoderm (adipose-like tissue, muscle-like tissue). Magnification 10×.

WT-iPS, SLE-iPS and ADPKD-iPS cell clones were allowed to spontaneously differentiate *in vitro*, through embryoid body cultures, and were assayed for the ability to differentiate into cells of the three embryonic germ layers. After 8 to 10 days culturing in suspension, embryoid bodies were plated on chamber slides and further cultured for 10 to 12 days. Immunostaining of differentiated cells with markers of three germ layers confirmed the presence of ectoderm (β-III-tubulin), endoderm (FOXA2) and mesoderm (CD31) cells (Figure 3). To determine the pluripotency of derived iPS cell clones *in vivo*, WT-iPS, SLE-iPS and ADPKD-iPS cells were injected subcutaneously into immunodeficient SCID-Beige mice and monitored for teratoma formation. iPS cell lines produced tumors 3 to 5 months post injection (Figure 4). Histological examination confirmed these tumors were teratomas with tissues of all three germ layers, including neuronal-rosette-like cells, gut tube-like structures, intestinal epithelial cells, adipose-like tissues and muscle-like structures (Figure 4). These results demonstrated that the renal disease-specific iPS cells possess hallmark properties of pluripotent stem cells.

### Autosomal mutation in the *PKD1 *gene verified in ADPKD-specific iPS cell clones

**Figure 5:**
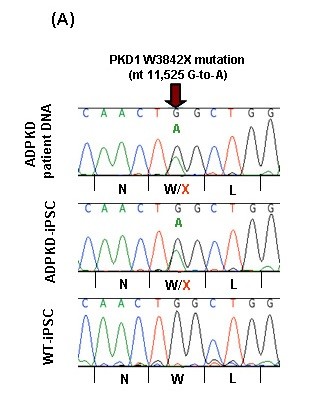
**Sequence analysis confirms *PKD1 *mutation in autosomal-dominant polycystic kidney disease-specific induced pluripotent stem cells**. Autosomal-dominant polycystic kidney disease (ADPKD) patient DNA and ADPKD- induced pluripotent stem (iPS) cell DNA were analyzed by sequencing analysis. Sequence of exon 41 of *PKD1 *showing the 11,525G > A change resulting in W3842X in the ADPKD-iPS cells (center) and the patient's germline DNA (top) but not in the Wilms tumor (WT)-iPS cells (bottom).

Our previous study has identified the heterozygous W3842X mutation in the *PKD1 *gene in the ADPKD patient (family 300004). We therefore tested whether ADPKD-iPS cell clones from this patient retained heterozygous W3842X mutation. As shown in Figure 5, sequence analysis of the *PKD1 *gene in the ADPKD-iPS and WT-iPS cell clones confirmed the heterozygous W3842X mutation only in the ADPKD-iPS cell clone, demonstrating that cellular reprogramming did not affect the autosomal mutation in the *PKD1 *gene.

## Discussion

Autologous, patient-specific iPS cells could attain customized tissue engineering and immunosuppression-free cell therapy for various diseases. For clinical applications, it is critical to derive iPS cells under a US Food and Drug Administration-compliant process. Although efficient and rapid generation of iPS cells from juvenile human primary keratinocytes was demonstrated through transduction with OCT4, SOX2, KLF4 and c-MYC [27], the feasibility and reproducibility of patient-specific iPS cell derivation from dermal keratinocytes required further evaluation. In the present article we demonstrate generation of kidney disease-specific iPS cells from skin keratinocytes. Importantly, skin biopsies were processed and expanded in animal-component-free reagents, and keratinocytes were reprogrammed under feeder-free conditions and expanded in serum-free media. Except for Matrigel, which is derived from a mouse cell line and used to coat culture plates for iPS cell culture, no animal component-containing reagent was used for iPS cell culture. Recently, fully defined adherent culture substrates composed of synthetic or recombinant molecules were found to support efficient human ES cell and iPS cell expansion [28-30]. A combination of feeder-free reprogramming of skin keratinocytes and one of these defined coating substrates would thus allow Good manufacturing practice-compliant iPS cell derivation, which represents an important step towards clinical iPS cell applications.

Important factors that may influence propagation of skin-derived cells and their nuclear reprogramming are: patient-specific cell survival defects; variations in age, gender and ethnic backgrounds; and chronic use of various medications. In this study, we reprogrammed skin-derived keratinocytes from patients with kidney transplantation on immunosuppressants, including mycophenolate, prednisone and tacrolimus. We were successful in propagating keratinocytes and in generating iPS cells from all three patients, demonstrating that age, disease backgrounds including ADPKD, SLE and WT, and chronic use of immunosuppressive drugs (especially antiproliferative mycophenolate) have no effect on skin cell propagation and iPS cell generation. Moreover, cellular reprogramming efficiency was comparable between keratinocytes from patients with ESRD and keratinocytes from healthy donors (data not shown). Our results thus indicate the feasibility of iPS cell derivation from patients with history of ESRD or maintained on chronic immunosuppression. Interestingly however, expansion of fibroblasts from skin biopsy samples collected from these patients was problematic. Active cell division stopped after few passages, most probably due to senescence. Therefore, we cannot rule out the possibility that long-term mycophenolate treatment might impair the capacity of fibroblast propagation *in vitro*. Further study will answer the potential impact of immunosuppressive drugs on the growth of skin-derived fibroblasts. In this regard, keratinocytes appeared to be a promising somatic cell source for patient-specific iPS cell derivation, particularly when patients have been treated with immunosuppressive drugs.

Integration of retroviral and lentiviral vectors into the host genome inherently harbors the risk of increased tumorigenicity due to insertional mutagenesis [31,32]. In addition, integration of reprogramming vectors could lead to sustained expression or reactivation of reprogramming factors, including proto-oncogene c-MYC. Expression of reprogramming factors could prevent proper differentiation of iPS cells, while reactivation of c-MYC expression can increase the risk of tumorigencity *in vivo *[33]. In our study, we used lentiviral vectors for reprogramming, where pluripotency factors were driven by a retroviral SFFV promoter. Although previous studies have demonstrated efficient suppression of a retroviral promoter in pluripotent stem cells [14], due to the pluripotency-associated factor TRIM28 [34], we found sustained transgene-specific transcripts in most of the patient-specific iPS cell clones tested. This was unexpected as we previously saw strong suppression of transgene expression in BJ fibroblast-derived iPS cell clones (data not shown). It is possible that this discrepancy was due to the difference in somatic cell sources used for reprogramming (fibroblasts vs. keratinocytes), or was simply due to clonal variations. To avoid the issues associated with the use of integrating vectors, it would be necessary to reprogram patient cells without using integrating vectors, especially for future clinical applications. Notably, various reports have demonstrated the possible cellular reprogramming strategies without using integrating vectors, including adenoviral vectors, episomal vectors or introduction of reprogramming proteins, RNAs or miRNAs [35-41]. We have recently reprogrammed patient's somatic cells using nonintegrating Sendai viral vectors and generated genomic modification-free, disease-specific iPS cells, and are currently in the process of analyzing the differences between iPS cells made with lentiviral vectors and nonintegrating Sendai vectors.

iPS cell technology enables the generation of patient-specific pluripotent stem cells that carry patient/disease-associated genotypes. Disease-specific iPS cells have been produced from various diseases including amyotrophic lateral sclerosis, Parkinson's disease, type 1 diabetes and Down's syndrome [20,21]. These clones provide unique opportunities to study disease-specific pathogenesis *in vitro *and *in vivo*. For instance, amyotrophic lateral sclerosis patient-specific iPS cells were successfully directed to differentiate into motor neurons, the cell type destroyed in amyotrophic lateral sclerosis [20]. iPS cells from autosomal kidney disease, such as ADPKD, would be useful for disease modeling, drug discovery and, eventually, autologous cell replacement therapies. We were the first to demonstrate successful iPS cell derivation from an ADPKD patient. As predicted, all ADPKD patient-derived iPS cells retained the W3842X mutation in the *PKD1 *gene. As these cells were not derived from cystic epithelium, we would not expect them to have further somatic mutations of *PKD1 *that may be important for cyst development. Differentiation of these iPS cells into collecting duct epithelial cells would allow their characteristics to be compared with wild-type cells.

Although renal transplantation has proven successful in treating patients with ESRD, the therapy is hampered by the shortage of donor organs. Differentiation of pluripotent stem cells into specific renal cell types, or possibly a transplantable whole kidney, would enable the potential of cell therapy for ESRD. Due to its complex structure and function, however, the kidney remains one of the most challenging organs to reconstruct. Extensive studies have established a method to efficiently induce mouse ES cells into renal progenitors and fully differentiated renal cells. For instance, treatment of mouse ES cells with the combinations of hepatocyte growth factor, activin A and Wnt4 [42], activin A, BMP-7 and retinoic acid [43], activin alone [44], or all of the four factors [45] has been shown to induce formation of intermediate mesoderm. Notably, derived intermediate mesoderm formed tubule-like structures in developing mouse kidney [42-44]. Although directed differentiation of human ES cells into renal lineage expressing lineage-specific markers has been reported using multiple growth factors [46,47], renal differentiation of human pluripotent stem cells needs further evaluation. Once an efficient differentiation protocol is established to differentiate human pluripotent stem cells into functional renal tissues, autologous iPS cells could bring in a novel, autologous kidney replacement therapy.

## Conclusions

Taken together our results demonstrate successful generation of keratinocyte-derived iPS cells from patients after kidney transplant maintained on chronic immunosuppression. Our results also indicate the feasibility of renal disease-specific iPS cell derivation from patients with a single gene mutation, such as ADPKD.

## Abbreviations

ADPKD: autosomal-dominant polycystic kidney disease; DMEM: Dulbecco's modified Eagle's medium; ES: embryonic stem; ESRD: end-stage renal disease; GAPDH: glyceraldehyde-3-phosphate dehydrogenase; H & E: hematoxylin and eosin; iPS: induced pluripotent stem; miRNA: microRNA; PBS: phosphate-buffered saline; PCR: polymerase chain reaction; PKD: polycystic kidney disease; RT: reverse transcription; SLE: systemic lupus erythematosus; WT: Wilms tumor.

## Competing interests

The authors declare that they have no competing interests.

## Authors' contributions

TT was responsible for conception and design, data collection, assembly, analysis and interpretation, manuscript writing, and final approval of the manuscript. PCH and YCK were responsible for conception and design, data analysis and interpretation, and final approval of the manuscript. ASA, JGDL, RE, YKK, TS, SO and JLS were responsible for collection and assembly of data, and final approval of the manuscript. YI was responsible for conception and design, data collection, assembly, analysis and interpretation, manuscript writing, and final approval of the manuscript.

## Supplementary Material

Additional file 1 **Antibodies and primer sequences**. Supplementary Table 1 presenting primary and secondary antibodies used in this study, and Supplementary Table 2 presenting primer sequences. Click here for file

Additional file 2 **Supplementary Figure 1 showing patient-specific iPS cells stained with control antibodies and ADPKD iPS cells stained with isotype control mouse (Ms) IgG, rabbit (Rb) IgG, secondary antibody FITC-conjugated mouse IgG and FITC-conjugated rabbit IgG**. Click here for file
