# Supplementary material for: Successful disease-specific induced pluripotent stem cell generation from patients with kidney transplantation
Source: Stem Cell Res Ther. 2011 Dec 6;2(6):48. doi: 10.1186/scrt89 (PMC3340557; doi:10.1186/scrt89)
Supplement: Additional file 1 — Antibodies and primer sequences. Supplementary Table 1 presenting primary and secondary antibodies used in this study, and Supplementary Table 2 presenting primer sequences. [file scrt89-S1.DOC]

**Supplementary Table 1.** Primary and secondary antibodies

| **Antibody** | **Company** | **Catalog No.** |
| --- | --- | --- |
| SSEA1 | Millipore | SCR001 |
| SSEA4 | Millipore | SCR001 |
| TRA-1-60 | Millipore | SCR001 |
| TRA-1-81 | Millipore | SCR001 |
| OCT3/4 | Cell Signaling Technology | #2750 |
| SOX2 | Cell Signaling Technology | #2748 |
| KLF4 | Abcam | ab26648 |
| NANOG | Abcam | Ab21624 |
| Beta-III-Tubulin | Abcam | Ab41489 |
| CD31 | Santa Cruz Biotechnology | SC1506 |
| HNF3 beta/FOXA2 | Millipore | #07-633 |
| FITC conjugated donkey-anti-rabbit IgG | Jackson Laboratories | #711-095-152 |
| FITC conjugated donkey-anti-mouse IgG | Jackson Laboratories | #715-095-151 |
| Texas Red conjugated donkey-anti-rabbit IgG | Jackson Laboratories | #711-075-152 |
| FITC conjugated donkey-anti-chicken IgG | Jackson Laboratories | #703-095-155 |
| Negative control for mouse primary antibodies | Dako | N1698 |
| Rabbit IgG | Jackson Laboratories | 011-000-003 |

**Supplementary Table 2.**  Primer Sequences

| **Gene** | **Forward Sequence** | **Reverse Sequence** | **Accession number** |
| --- | --- | --- | --- |
| OCT4 | AGCGAACCAGTATCGAGAAC | TTACAGAACCACACTCGGAC | BC117435.1 |
| SOX2 | AGCTACAGCATGATGCAGGA | GGTCATGGAGTTGTACTGCA | BC013923.2 |
| NANOG | TGAACCTCAGCTACAAACAG | TGGTGGTAGGAAGAGTAAAG | AB093576.1 |
| c-MYC | ACTCTGAGGAGGAACAAGAA | TGGAGACGTGGCACCTCTT | BC000141 |
| KLF4 | TCTCAAGGCACACCTGCGAA | TAGTGCCTGGTCAGTTCATC | BC029923.1 |
| hTERT | TGTGCACCAACATCTACAAG | GCGTTCTTGGCTTTCAGGAT | AB085628.1 |
| GDF3 | AAATGTTTGTGTTGCGGTCA | TCTGGCACAGGTGTCTTCAG | AF263538.1 |
| OCT4 transgene | CTCCGACAGACTGAGTCGCCCGGG | CCTTGAGGTACCAGAGATCT |  |
| c-MYC  transgene | CTCCGACAGACTGAGTCGCCCGGG | CGCTCGAGGTTAACGAATT |  |
| KLF4 transgene | CTCCGACAGACTGAGTCGCCCGGG | CCTTGAGGTACCAGAGATCT |  |
| GAPDH | AGC CAC ATC GCT CAG ACA CC | GTA CTC AGC GGC CAG CAT CG | BT006893.1 |
