# Supplementary figures and images for: Successful disease-specific induced pluripotent stem cell generation from patients with kidney transplantation
Source: Stem Cell Res Ther. 2011 Dec 6;2(6):48. doi: 10.1186/scrt89 (PMC3340557; doi:10.1186/scrt89)

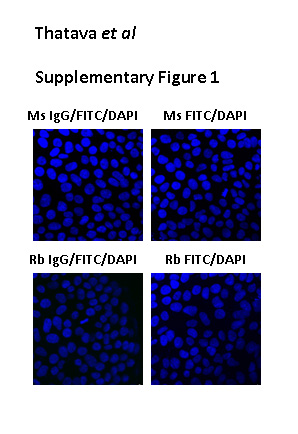

Supplement: Additional file 2 — Supplementary Figure 1 showing patient-specific iPS cells stained with control antibodies and ADPKD iPS cells stained with isotype control mouse (Ms) IgG, rabbit (Rb) IgG, secondary antibody FITC-conjugated mouse IgG and FITC-conjugated rabbit IgG. [file scrt89-S2.JPEG]
